# Validation of the Croatian version of CIVIQ quality of life questionnaire in patients with chronic venous disorders

**DOI:** 10.3325/cmj.2017.58.292

**Published:** 2017-08

**Authors:** Tamara Sinožić, Ksenija Baždarić, Dina Šverko, Alen Ružić, Milica Katić

**Affiliations:** 1 Family Medicine Office, Mošćenička Draga, Croatia; 2 Department of Medical Informatics, Rijeka University Faculty of Medicine, Rijeka, Croatia; 3 Department of Internal Medicine, Rijeka University Faculty of Medicine, Rijeka, Croatia; 4 Department of Family Medicine, Zagreb University School of Medicine, Zagreb, Croatia

## Abstract

**Aim:**

To test the psychometric properties of the Croatian version of the Chronic Venous Insufficiency Quality of Life (CIVIQ) Questionnaire and to assess the quality of life in patients with chronic venous disorders of all stages.

**Methods:**

This cross-sectional study performed between 2014 and 2015 in a private family practice assessed the factorial validity, cross-sectional validity, and reliability of the Croatian CIVIQ 20-item questionnaire completed by 428 adult patients (78% women) with chronic venous disorders classified according to the Clinical-Etiologic-Anatomic-Pathophysiologic (CEAP) C classification as stages C1-C6.

**Results:**

Median patient age was 52 years (5th-95th percentile, 30-77). The distribution according to the clinical stages of chronic venous disorders was as follows: C1 (n = 78, 18%), C2 (n = 192, 45%), C3 (n = 53, 12%), C4 (n = 44, 10%), C5 (n = 13, 3%), and C6 (n = 48, 11%). The CIVIQ-20 factorial structure was unstable, and six items were excluded from the analysis to test the psychometric properties of the shortened version (CIVIQ-14). CIVIQ-14 has three dimensions (physical, psychological, and pain). Internal consistency reliability is high for the entire CIVIQ-14 (Cronbach α = 0.92) and for all CIVIQ-14 dimensions (α≥0.80). The median quality of life significantly decreased with higher CEAP C stages as follows: C1/C2 (86, 50-100); C3/C4 (75, 36-98); C5/C6 (67, 31-95) (*P* < 0.001). Post-hoc analysis showed a higher quality of life in C1/C2 than in other groups (*P* < 0.001).

**Conclusion:**

The shortened CIVIQ-14 version is useful for assessing the quality of life in patients with chronic venous disorders in everyday clinical practice. To achieve a stable validated instrument, we recommend a cross-cultural validation of items that have loadings on more than one factor.

Chronic venous disorders (CVDs) develop through a cascade of events that lead to pathological changes in the venous walls and/or valves and eventually to vein hypertension (1). The most common early symptoms include leg pain, heaviness, tiredness, stinging, itching, cramps, restlessness, and swelling (2). The CVDs are classified in the 2004 revision of the American Venous Forum Clinical-Etiologic-Anatomic-Pathophysiologic (CEAP) classification system according to clinical signs and symptoms, etiology, anatomical classification, and pathophysiology. The clinical classification includes seven coded stages, C0 to C6. Specifically, C0 means that there are no visible or palpable signs of venous disease, C1 denotes telangiectasias or reticular veins, C2 varicose veins, C3 edema, C4 skin changes (C4a- hyperpigmentation or eczema and C4b-lipodermatosclerosis or atrophie blanche), C5 healed venous ulcer, and C6 active venous leg ulcer (3).

The worldwide prevalence of CVDs in the adult population as assessed by the Vein Consult program is 81%, ie, 20% for stage C0s and 61% for stages C1-C6 (C1 21.6%, C2 16%, C3 14.6%, C4 7.1%, C5 1.4%, and C6 0.5%) (4). With such a high prevalence, CVDs greatly burden the society and affect the quality of life (QoL) of patients and their families (5). Data on CVDs prevalence are lacking for the population of Croatia, because all venous diseases are reported under the overarching International Classification code ICD I80-I89 – Diseases of veins, lymphatic vessels, and lymph nodes, not elsewhere classified.

Current research indicates that higher CVDs stages are associated with lower QoL and that treatment significantly improves QoL (2,6,7). In addition, QoL is lower in patients who are elderly, overweight, or had previous superficial or deep vein thrombosis (7). In patients with venous leg ulcers, the disease affects all aspects of life from physical and psychological to social (8). QoL is usually measured with general or disease-specific questionnaires. The most common general questionnaires are the 36-Item Short Form Health Survey (SF-36), the 12-Item Short Form (SF-12) – both part of the Medical Outcomes Study – and the EuroQoL five dimensions questionnaire (EQ-5D) (9). One of the most common disease-specific questionnaires for CVDs is the Chronic Venous Insufficiency Questionnaire (CIVIQ), whose original French version consists of 20 items (10). CIVIQ-20 has been translated into a number of languages (mostly by forward-backward translation) and psychometrically validated (11-14). The Croatian version of CIVIQ-20 is a forward-backward translation that has not been validated psychometrically in terms of reliability and factorial and cross-sectional validity. Furthermore, it has not yet been used to assess QoL in patients with healed venous ulcers (C5) and active venous leg ulcers (C6).

The aim of this study was to validate the Croatian version of CIVIQ-20 by determining its psychometric dimensions and, at the same time, to determine QoL in patients with all CVDs stages.

## PATIENTS AND METHOD

### Patients

Patients with CVDs were recruited in a private family practice between 15 September 2014 and 15 September 2016.

Chronic venous disorder was diagnosed based on the reported symptoms and clinical examination findings and classified according to the CEAP stages C1 to C6. All patient data, including age, sex, and body mass index (BMI) were entered into electronic health record. BMI was defined as follows: 20-24.99 – normal; 25-29.99 – overweight, 30-34.99 – obese, and >35 – severely obese.

### Questionnaire

The original CIVIQ-20 version has 20 items and four dimensions including pain (4 items), physical dimension (4 items), psychological dimension (9 items), and social dimension (3 items), all scored on the Likert scale from 1 (no symptom, sensation or trouble) to 5 (highest intensity or frequency, depending on the item). For each completed questionnaire, we calculated the Global Index Score (GIS) following the instructions available at the official CIVIQ web page (14,15). All scores were calculated as ([Final score – minimal possible score]/[Theoretical maximal – minimal score]) × 100. The GIS CIVIQ-20 was calculated as ([Final score – 20]/80) × 100. The minimal score for CIVIQ 20 was 20, maximum 100. The GIS CIVIQ-14: ([Final score – 14] / 56) × 100. The minimum score for CIVIQ-14 was 14, and maximum 70. For each dimension or factor, the score was calculated accordingly, depending of the number of items and minimum and maximum scores.

The patients were asked to answer the questionnaire at the first visit.

### Statistical analysis

Categorical variables were summarized as absolute and relative frequencies. Continuous variables were summarized as median and 5th-95th percentile. Kolmogorov-Smirnov test was used to test distribution normality. Correlation between variables was calculated with Spearman's correlation coefficient.

Factorial validity of the Croatian CIVIQ-20 version was evaluated with exploratory factor analysis (EFA), but before factorial analysis we ran the Kaiser-Meyer-Olkin (KMO) measure of sampling adequacy and the Bartlett's test of sphericity to test the suitability of the item correlation matrix for factoring. In EFA, we used principal axis factoring (PAF) as the factor extraction method and Promax as the rotation method. We included the extracted factors with the eigenvalue >1, which accounted for more >10% of the variance, and which passed visual inspection on the scree plot. The factor analysis procedure use the pattern of correlation between questionnaire items, which represent directly measured manifest variables, grouping them by the variance they share which is captured by factors that are interpreted as latent dimensions, inferred constructs that are not directly measured. Consequently, each extracted factor or dimension is defined only by questionnaire items to which it relates (16).

To assess the cross-sectional validity we used nonparametric statistics, because score distributions were not normal. For the GIS and the scores for three dimensions (15), we used the Kruskal-Wallis test to test for the differences between patient groups according to the CEAP classification. For *post-hoc* comparisons we used the Mann-Whitney U test. Cronbach's alpha was used as a measure of internal consistency reliability. *P* < 0.05 was considered significant. The data were analyzed with the SPSS 16.0 (SPSS Inc., Chicago, IL, USA) and MedCalc statistical software, version 11.2.0.0 (MedCalc Inc., Mariakerke, Belgium).

## RESULTS

### Patient characteristics

Of 428 patients, 332 (78%) were women. The median age of all patients was 52 years (range 19-93). More than a third (n = 149, 35%) had a normal BMI, 190 (44%) were overweight, and 89 (21%) were obese or severely obese.

In 192 (45%) patients, the disorder was classified as C2, in 78 (18%) as C1, in 53 (12%) as C3, in 44 (10%) as C4, in 13 (3%) as C5, and in 48 (11%) as C6. The CVDs stages significantly correlated with BMI (r_s_ = 0.54, *P* < 0.001).

### Acceptability

The floor and ceiling effects were assessed to evaluate score distribution of the individual items. The items 14, 18, and 20 demonstrated a substantial ceiling effect with more than 70% of the respondents having the lowest score: item 14 (n = 346, 80%), item 18 (n = 324, 75%), and item 20 (n = 336, 78%). No floor effects were observed.

### Construct validity

#### Factorial validity

The KMO value (0.97) and the Bartlett’s test of sphericity (*P* < 0.001) indicated that factor analysis is appropriate for these data.

Principal Axis Factoring (PAF) analysis yielded a four-factor model. The eigenvalues of the four extracted factors were 10.11 for the physical dimension, 1.60 for the psychological dimension, 1.05 for pain, and 0.83 for the social dimension. These four factors accounted for 60% of the common variance. The structure matrix (correlations of each item with the extracted dimensions) and the obtained factorial structure (1st EFA) was complex rather than simple (Table 1). Eight items including items 2, 4, 7, 9, 10, 12, 13, and 15 had high loadings (>0.40), ie, correlations with factor, on more than one extracted factor.

**Table 1:** Structure matrices (correlations of each item with the extracted dimensions) of the Chronic Venous Insufficiency Quality of Life Questionnaire (CIVIQ-20) factor analysis

CIVIQ-20 item	CIVIQ-20 dimensions (factor loadings)						
	1st EFA*				2nd EFA*		
	physical	psychological	pain	social	physical	psychological	pain
1. Leg pain	0.39	0.14	0.79	0.07	0.39	0.13	0.75
2. Work or daily activities impairment	0.46	0.26	0.76	0.14	0.46	0.26	0.75
3. Slept badly	0.34	0.21	0.72	0.15	0.34	0.22	0.72
4. Standing for a long time	0.51	0.24	0.57	0.31	0.52	0.26	0.62
5. Climbing several floors	0.82	0.24	0.37	0.09	0.82	0.23	0.36
6. Crouching/kneeling down	0.78	0.25	0.36	0.03	0.78	0.23	0.34
7. Walking at brisk pace	0.77	0.26	0.40	0.10	0.77	0.25	0.39
8. Traveling by car	0.38	0.28	0.40	0.35	0.40	0.32	0.45
9. Performing household tasks	0.55	0.30	0.50	0.41	0.56	0.34	0.56
10. Going out for the evening, wedding party, cocktail	0.60	0.28	0.37	0.41	0.61	0.32	0.44
11. Playing a sport, exerting physically	0.71	0.29	0.36	0.32	0.71	0.32	0.41
12. Feeling nervous/tense	0.40	0.49	0.57	0.08	0.40	0.48	0.55
13. Feeling tired quickly	0.61	0.43	0.42	0.20	0.62	0.44	0.43
14. Feeling of being a burden	0.38	0.56	0.27	0.07	0.39	0.56	0.25
15. Being cautious all the time	0.51	0.41	0.37	-0.04	0.50	0.39	0.33
16. Feeling embarrassed about showing own legs	0.07	0.44	0.16	0.27	0.10	0.47	0.20
17. Being irritated easily	0.33	0.66	0.31	0.16	0.34	0.67	0.32
18. Feeling handicapped	0.31	0.64	0.22	0.16	0.32	0.66	0.23
19. Finding hard to get going in the morning	0.37	0.67	0.32	0.06	0.38	0.66	0.30
20. Having no desire to go out	0.37	0.71	0.12	-0.06	0.37	0.68	0.08

*EFA – exploratory factor analysis.

The first factor, which can be interpreted as the physical dimension, highly correlated (>0.50) with all four items – items 5, 6, 7, and 9 – from the original French CIVIQ-20. However, four other items, specifically item 10 – “going out for the evening, weddings, party, cocktail”, item 11 – “playing a sport, exerting physically”, item 13 – “feeling tired quickly”, and item 15 – “being cautious all the time” had the highest correlations with the physical dimension. Items 10 and 11 were originally intended to measure the social dimension and items 13 and 15 the psychological dimension.

The second extracted factor was the psychological dimension, with the highest loadings for six of the nine original French CIVIQ-20 items, ie, items 12-19 and 20. Item 12 (“feeling nervous/tense”), showed the highest loading on the pain dimension and items 13 (“feeling tired quickly”) and 15 (“being cautious all the time”) had the highest loadings on the physical dimension.

The third factor, pain (original items 1, 2, 3, and 4 in the French CIVIQ-20), had all four items from the original definition and two additional items: item 8 (“travelling by car”), originally intended to measure the social dimension, and item 12 (“feeling nervous/tense”), originally intended to measure the psychological dimension.

The fourth extracted factor was originally meant to be a social dimension, which included items 8, 9, and 10. The correlations between the factor and the items did not exceed 0.41, and none of the items had a higher correlation with the fourth factor than with other factors.

Because of the complex factorial structure, lack in extracting an interpretable fourth factor, the criterion of the eigenvalue higher than 1 (to consider extracted factor significant), visual inspection of the scree plot, and similarity between our findings and the EFA results of the translated versions of CIVIQ-20, we performed the second explanatory factor analysis.

This second EFA was a PAF analysis with Promax rotation and a three-factor model (Table 1). The first, second, and the third factor can be interpreted as physical, psychological, and pain dimensions, respectively. These factors explained 58% of the common item variance. Most of the items showed a correlation pattern with these three factors, which was similar to the original French CIVIQ-20. However, the factors were still mixed and some of the items showed complexity.

In an attempt to achieve a stable structure of the Croatian version of CIVIQ-20, we then eliminated six items including items 4, 8, 9, 13, 15, and 19 from the questionnaire, as Launois et al (17) did for the French CIVIQ-14 version, and then factorized the Croatian version of CIVIQ-14 and ran PAF with Promax rotation using the three-factor model (Table 2). KMO (0.93) and the Bartlett's test of sphericity (*P* < 0.001) confirmed the adequacy of the correlation matrix for factor analysis. The eigenvalues of the three extracted factors were 7.04 (physical dimension), 1.45 (psychological dimension), and 1.01 (pain). These three factors accounted for 59% of the common variance.

**Table 2:** Structure matrix (correlations of each item with the extracted dimensions) of the factor analysis of the Chronic Venous Insufficiency Quality of Life Questionnaire (CIVIQ-14)

CIVIQ-14 item	CIVIQ-14 dimensions (factor loadings)		
	physical	psychological	pain
1. Leg pain	0.40	0.13	0.78
2. Work or daily activities impairment	0.48	0.26	0.75
3. Slept badly	0.36	0.21	0.73
4. Climbing several floors	0.83	0.23	0.35
5. Crouching/kneeling down	0.78	0.23	0.35
6. Walking at brisk pace	0.79	0.23	0.40
7. Going out for the evening, wedding party, cocktail	0.60	0.32	0.41
8. Playing a sport, exerting physically	0.73	0.33	0.39
9. Feeling nervous/tense	0.40	0.48	0.56
10. Feeling of being a burden	0.39	0.55	0.26
11. Feeling embarrassed about showing own legs	0.10	0.49	0.18
12. Being irritated easily	0.35	0.66	0.32
13. Feeling handicapped	0.33	0.70	0.22
14. Having no desire to go out	0.38	0.64	0.11

#### Cross-sectional validity

Cross-sectional clinical validity of CIVIQ-14 was verified by testing the assumption that patients with higher CVDs stage would report lower QoL than patients with lower stage and that patients with higher BMI will have lower QoL. To test the group differences, we merged patient groups C1 and C2, C3 and C4, and C5 and C6 to obtain comparable group sizes and a more even distribution of patients per group.

Our findings confirmed the assumption that QoL decreased with increase in CVDs stage (χ^2^ = 43.76, *P* < 0.001; Table 3). GIS score and scores in all three dimensions in the C1 and C2 group were significantly higher than scores in other two groups (*P* < 0.001).

**Table 3:** Chronic Venous Insufficiency Quality of Life Questionnaire (CIVIQ-14) scores according to the Clinical-Etiologic-Anatomic-Pathophysiologic (CEAP) C classification*

CIVIQ-14	CEAP C (median, 5th-95th percentile)			*P*
	C1 and C2	C3 and C4	C5 and C6	
No. (%) of patients	270 (63.0)	97 (23.0)	61 (14.0)	
Global index score	86 (50-100)*	75 (36-98)	67 (31-95)	<0.001
Pain	75 (33-100)*	67 (16-100)	58 (0-92)	<0.001
Physical dimension	87 (37-100)*	69 (25-100)	63 (25-100)	<0.001
Psychological dimension	87 (54-100)*	83 (33-100)	83 (46-96)	<0.001

*Statistically different from the other two groups at *P* < 0.001.

Also, the CVDs stage (CEAP C) and BMI negatively correlated with the QoL (GIS) and its dimensions (Table 4).

**Table 4:** Correlation of Clinical-Etiologic-Anatomic-Pathophysiologic (CEAP C) classification and its dimensions, body mass index (BMI), and quality of life according to global index score (GIS)

	CEAP C	BMI	GIS	Pain	Physical dimension	Psychological dimension
CEAP C						
r_s_*	-	0.54	-0.33	-0.27	-0.33	-0.21
*P*		<0.001	<0.001	<0.001	<0.001	<0.001
n		428	428	428	428	428
BMI						
r_s_	-	-	-0.24	-0.18	-0.30	-0.11
*P*			<0.001	<0.001	<0.001	0.002
n			428	428	428	428
GIS						
r_s_	-	-	-	0.81	0.89	0.81
*P*				<0.001	<0.001	<0.001
n				428	428	428
Pain						
r_s_	-	-	-	-	0.68	0.55
*P*					<0.001	<0.001
n					428	428
Physical dimension						
r_s_	-	-	-	-	-	0.60
*P*						<0.001
n						428

*r_s_ – Spearman coefficient of correlation.

### Reliability

#### Internal consistency

Cronbach's alpha of overall CIVIQ-20 was 0.94 and of CIVIQ-14 0.92. The reliability coefficients of the CIVIQ-14 dimensions were lower than those of the global scales. Cronbach's α was 0.86 for the pain dimension (items 1 to 3), 0.86 for the physical dimension (items 4 to 8), and 0.80 for the psychological dimension (items 9 to 14).

The mean inter-item correlation for the entire CIVIQ-14 was 0.45, and for the psychological, physical, and pain dimensions 0.45, 0.66, and 0.67, respectively.

## DISCUSSION

Our validation procedure of the Croatian version of CIVIQ-20 corresponds to the procedures and results from other countries. The pattern of correlations between the first three extracted factors and the questionnaire items in our study is similar to that reported by Launois et al (10) with the original French CIVIQ-20 and in the Polish, Czech, and Spanish versions (17). In all these versions, including the Croatian, the factor intended to measure the social dimension was lost in validation, probably due to a poor definition of the dimension in the initial design of the questionnaire, as it had only three items (16). It seems that, against the initial assumption, the social dimension could not apply to patients with CVDs stages C1 to C4, as they were unlikely to report difficulties in social functioning, unlike higher-stage patients (C5 and C6).

Because of this structural instability of CIVIQ-20 and consequent inapplicability of total scores across dimensions, Launois et al (17) removed six items that contributed the most to structural instability and obtained a more stable version of the questionnaire known as CIVIQ-14. We repeated the procedure with the Croatian CIVIQ-20 version and the remaining 14 items had pattern nearly identical to the original CIVIQ-14, except for the instability in item 9 (“feeling nervous/tense”). The item was unstable in other validations as well. For example, in a recent validation study of CIVIQ-14 in 17 countries, the same item (“feeling nervous/tense”) had loadings on the psychological and pain dimensions (18). The instability of item 9 lies in its construct, because it measures not only a psychological symptom (feeling nervous), but also a physical one (feeling tense). Also, because pain is the consequence of tension, the item has significant loadings on all three factors. We suggest that the word “tense” (“napeto” in Croatian) should be left out in future versions of CIVIQ-14 in order to obtain a more stable factorial structure and limit the use to the psychological dimension alone. Apart from item 9, two more items (items 6 and 7) in our validation had loadings on more than one factor. Similar results were found in the Czech validation of CIVIQ-14, where item 7 (“going out for the evening, parties...”) had loadings on pain and physical dimensions (18). The validation study in Serbia also revealed three items with loadings on both physical dimension and pain (13). Therefore, we believe that cross-cultural adaptation would be welcome in the Croatian version, especially for the items where pain and physical dimension are combined, such as items 6 and 7, as they have loadings on both factors.

Our findings confirmed that CIVIQ-14 is a reliable instrument with very high internal consistency of the whole scale (Cronbach's α = 0.92) and of its three dimensions (pain, physical, and psychological dimension; Cronbach's α≥0.80). Similar results were reported in other validation studies (11-14).

Our results also confirmed that patients with higher clinical stages of CVDs report lower overall QoL and lower scores for all three dimensions. This suggests that CIVIQ-14 and its dimensions are reliable clinical indicators that may help in everyday practice. Again, similar results were reported in the Serbian validation study (13), where the measured QoL in their patients was lower than in our study in regard to the CVDs stage. This may point to factors other than those measured by either CIVIQ-14 or CIVIQ-20, such as gross national product, poverty, and related issues, such as poor health care or patient self-care.

The patients in our study had the lowest scores on the pain dimension. These results suggest that pain, as part of CVDs, is related mostly to the general perception of the quality of life. We believe that the experience of pain was far more salient in comparison to the physical and psychological problems captured by other two dimensions of CIVIQ-14. Therefore, pain was probably the main reason why patients sought medical consultation. While pain and physical dimension scores rose from one CVDs stage to the next for about 6-8 points, the psychological dimension reached plateaus at stages C3/C4. It seems that patients with the CVD stages C5/C6 adapted to the disease over time.

Our study included patients with C6 stage. Until now, C6-patients have not been included in the international validation (11), save for Serbia (13) and Turkey (19).

The correlation of CVDs stage with the overall QoL was negative, as it was with all three dimensions. The correlation was negative between BMI and overall QoL and positive between BMI and CVD stage. This was expected due to reports of a significantly higher risk of severe CVDs in overweight patients (7).

We did not compare the CIVIQ questionnaire with other general or specific QoL measures, as it was done many times earlier. However, our findings are somewhat limited because we did not follow-up our patients and retest the reliability of the questionnaire. This we plan to do in a future study.

In conclusion, the CIVIQ-14 questionnaire is a simple, brief, and effective tool for measuring the QoL in family practice patients with CVDs, which may complement clinical diagnosis. It is a valid and reliable measurement instrument, which takes five to ten minutes to complete during a visit, and can provide helpful information in everyday practice. It not only provides information about the affected population, but also about each patient, which allows for an individual approach to treatment.
